# Machine Learning Using Real-World and Translational Data to Improve Treatment Selection for NSCLC Patients Treated with Immunotherapy

**DOI:** 10.3390/cancers14020435

**Published:** 2022-01-16

**Authors:** Arsela Prelaj, Mattia Boeri, Alessandro Robuschi, Roberto Ferrara, Claudia Proto, Giuseppe Lo Russo, Giulia Galli, Alessandro De Toma, Marta Brambilla, Mario Occhipinti, Sara Manglaviti, Teresa Beninato, Achille Bottiglieri, Giacomo Massa, Emma Zattarin, Rosaria Gallucci, Edoardo Gregorio Galli, Monica Ganzinelli, Gabriella Sozzi, Filippo G. M. de Braud, Marina Chiara Garassino, Marcello Restelli, Alessandra Laura Giulia Pedrocchi, Francesco Trovo'

**Affiliations:** 1 Medical Oncology Department, Fondazione IRCCS Istituto Nazionale Tumori, 20133 Milan, Italy; roberto.ferrara@istitutotumori.mi.it (R.F.); claudia.proto@istitutotumori.mi.it (C.P.); giuseppe.lorusso@istitutotumori.mi.it (G.L.R.); giulia.galli@istitutotumori.mi.it (G.G.); alessandro.detoma@istitutotumori.mi.it (A.D.T.); marta.brambilla2@istitutotumori.mi.it (M.B.); mario.occhipinti@istitutotumori.mi.it (M.O.); sara.manglaviti@istitutotumori.mi.it (S.M.); teresa.beninato@istitutotumori.mi.it (T.B.); achille.bottiglieri@istitutotumori.mi.it (A.B.); giacomo.massa@istitutotumori.mi.it (G.M.); emma.zattarin@istitutotumori.mi.it (E.Z.); rosaria.gallucci@istitutotumori.mi.it (R.G.); edoardogregorio.galli@studenti.unimi.it (E.G.G.); monica.ganzinelli@istitutotumori.mi.it (M.G.); filippo.debraud@istitutotumori.mi.it (F.G.M.d.B.); marina.garassino@istitutotumori.mi.it (M.C.G.); 2 Department of Electronics, Information and Bioengineering, Politecnico di Milano, 20133 Milan, Italy; alessandro.robuschi@polimi.it (A.R.); marcello.restelli@polimi.it (M.R.); alessandra.pedrocchi@polimi.it (A.L.G.P.); francesco1.trovo@polimi.it (F.T.); 3 Tumor Genomics Unit, Department of Research, Fondazione IRCCS Istituto Nazionale dei Tumori, 20133 Milan, Italy; mattia.boeri@istitutotumori.mi.it (M.B.); gabriella.sozzi@istitutotumori.mi.it (G.S.)

**Keywords:** non-small cell lung cancer, immunotherapy, biomarker, artificial intelligence, machine learning

## Abstract

**Simple Summary:**

In this paper, the authors show that artificial intelligence (AI) and machine learning (ML) are useful approaches to integrate multifactorial data and helpful for personalized prediction. In detail, compared to PD-L1 for advanced non-small cell lung cancer (NSCLC), ML tools predicted better responder (R) and non-responder (NR) patients to immunotherapy (IO). It was also able to indirectly foresee OS and PFS of R and NR patients. Given the high incidence of NSCLC, and the absence of reliable biomarkers to predict the response to IO other than PD-L1, the authors believe this research may be of great interest to anyone involved in thoracic oncology. Furthermore, given the growing interest from the scientific community in AI and ML, the authors believe that this manuscript could represent a fascinating topic to anyone who needs to exploit the enormous potential of these tools in the treatment of cancer.

**Abstract:**

(1) Background: In advanced non-small cell lung cancer (aNSCLC), programmed death ligand 1 (PD-L1) remains the only biomarker for candidate patients to immunotherapy (IO). This study aimed at using artificial intelligence (AI) and machine learning (ML) tools to improve response and efficacy predictions in aNSCLC patients treated with IO. (2) Methods: Real world data and the blood microRNA signature classifier (MSC) were used. Patients were divided into responders (R) and non-responders (NR) to determine if the overall survival of the patients was likely to be shorter or longer than 24 months from baseline IO. (3) Results: One-hundred sixty-four out of 200 patients (i.e., only those ones with PD-L1 data available) were considered in the model, 73 (44.5%) were R and 91 (55.5%) NR. Overall, the best model was the linear regression (RL) and included 5 features. The model predicting R/NR of patients achieved accuracy ACC = 0.756, F1 score F1 = 0.722, and area under the ROC curve AUC = 0.82. LR was also the best-performing model in predicting patients with long survival (24 months OS), achieving ACC = 0.839, F1 = 0.908, and AUC = 0.87. (4) Conclusions: The results suggest that the integration of multifactorial data provided by ML techniques is a useful tool to select NSCLC patients as candidates for IO.

## 1. Introduction

Lung cancer is the leading cancer-related death worldwide with around 470,000 new cases and 390,000 deaths in Europe. Non-small cell lung cancer (NSCLC) is the most common histology for around 85% [1]. Until 2015, the median OS of patients with metastatic NSCLC was around 12 months [2]. The advent of immunotherapy (IO) has radically changed the treatment paradigm of many cancers including NSCLC, prolonging survival of metastatic patients from 12 to a median of around 24 months [2]. Some patients that better respond to IO reached longer survival of up to or more than 5 years [3]. However, only 30–50% of patients will benefit from IO in the long term [4,5,6].

Currently in clinical practice, programmed death-ligand 1 (PD-L1) is the only biomarker used to predict IO response. However, its predictive performance is not satisfactory (around 30–50%) [7]. Beyond PD-L1, several other biomarkers have been identified and used to profile patient prediction, including tumor mutational burden (TMB) [8], tumor microenvironment (TME) [9], microRNA (miRNA) [10], immune gene signatures [11], gut microbiome [12], radiomics [13], and baseline clinical features or their combination in different scores [14,15].

Indeed, it is implausible that a single biomarker is able to profile prediction or prognosis with high accuracy, since the immune system displays dynamic complexity when interacting with its TME. To handle the density of the available data, artificial intelligence (AI) frameworks and, more specifically, machine learning (ML) techniques, provide efficient, pioneering, and theoretically sound approaches to construct decision-making tools providing individualized prediction [16].

Among molecular biomarkers, the plasma microRNA signature classifier (MSC), reflecting an immunosuppressive host status, was here considered [10]. It was previously trained in lung cancer screening cohorts to evaluate the individual risk to develop the aggressive form of the disease [17,18]. More recently, the MSC prognostic value was also validated in advanced NSCLC patients treated with single agent IO [19], and its combination with different clinical scores confirmed its independence from other prognostic features in this setting [20].

This study aimed to integrate real-world data and the MSC test to develop a machine learning algorithm to predict response to and efficacy of IO in NSCLC patients. The study also investigated the role of the MSC test and its added value to the algorithm prediction capability, given that this latter test is costly and still not included in standard clinical practice as a predictive/prognostic biomarker.

## 2. Materials and Methods

### 2.1. Study Population

From July 2015 to November 2020, we conducted a prospective observational study (Apollo, INT 22_15) enrolling 200 consecutive aNSCLC patients receiving single-agent anti-PD-(L)-1 inhibitors in first- (*n* = 70) or second- and further-line therapy (*n* = 130). Complete real-world data and whole blood samples were collected as per clinical practice. The MSC test was prospectively assessed in plasma samples collected at baseline IO.

Inclusion criteria were the following: cytological/histological diagnosis of advanced NSCLC, patients (relapsed or stage IIIB to IV) that had received at least one infusion of first- or further-line single-agent IO. Patients without baseline IO MSC test information were excluded from the study.

This prospective study was conducted at Fondazione IRCCS Istituto Nazionale Tumori of Milan in Italy in collaboration with Politecnico di Milano, for the data analytics. This study was approved by the ethical committee of Fondazione IRCCS Istituto Nazionale Tumori of Milan, and all included patients signed informed consent prior to plasma and data collection in accordance with the Declaration of Helsinki, Good Clinical Practice and local ethical guidelines.

### 2.2. Real World Data Collection: Clinical, Blood, and Tissue Data

For this study, demographic, medical history, tumor stage, PD-L1 (PD-L1 testing was mostly carried out using the PD-L1 IHC 22C3), molecular and radiological data, concomitant medications, treatment responses, and survival follow-up were collected and integrated to develop e new predictive model of IO response and efficacy in NSCLC.

### 2.3. Omic Collection: MSC Blood Test

Whole blood was collected in 10 mL K2EDTA Vacutainer tubes, and the plasma separated by two centrifugation steps. Total RNA was extracted from 200 μL plasma samples. MicroRNA expression was determined by quantitative reverse transcription PCR (RT-qPCR) as previously described [19,21].

**Figure 1:**
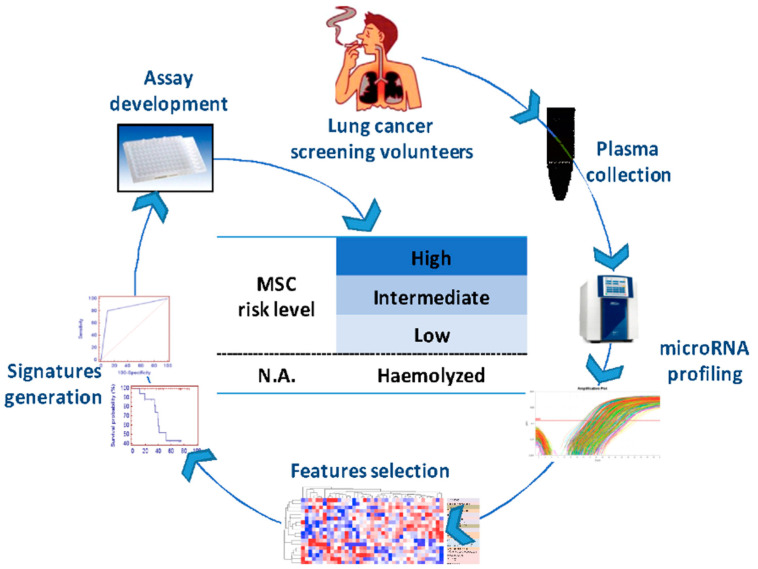
Development of the plasma MSC test using 24 miRNA and 4 risk groups. MSC: microRNA signature classifier; N.A.: not analyzable.

The MSC algorithm using 24 miRNAs defined four different classes of risk: low (L) intermediate (I) and high (H) risk [18] and highly hemolyzed (E). The fourth category E, thus not analyzable plasma samples, due to the unspecific release of miRNAs in the presence of blood cell lyses, was included [10] (Figure 1). The 24 miRNAs were hsa-miR-101-3p, hsa-miR-106a-5p, hsa-miR-126-3p, hsa-miR-133a-3p, hsa-miR-140-3p, hsa-miR-140-5p, hsa-miR-142-3p, hsa-miR-145-5p, hsa-miR-148b-3p, hsa-miR-15b-5p, hsa-miR-16-5p, hsa-miR-17-5p, hsa-miR-197-3p, hsa-miR-19b-3p, hsa-miR-21-5p, hsa-miR-221-3p, hsa-miR-28-3p, hsa-miR-30b-5p, hsa-miR-30c-5p, hsa-miR-320a, hsa-miR-451a, hsa-miR-486-5p, hsa-miR-660-5p, and hsa-miR-92a-3p. Patients with this category were previously observed to have an intermediate prognosis between patients with H and I risk [20].

### 2.4. Treatment Administration

IO was administered intravenously (IV) as monotherapy. Nivolumab was administered initially at a dose of 3 mg/kg and later, from May 2018 in Italy, at a fixed dose of 240 mg every 2 weeks (w). Pembrolizumab was administered at a fixed dose of 200 mg as first line and at a dose of 2 mg/kg every 3 weeks in second or third-line setting. Atezolizumab was administered at a fixed dose of 1200 mg every 3 weeks, and durvalumab at a dose of 10 mg/kg every 2 weeks.

Therapy was continued until progressive disease (PD), intolerable toxicity, withdrawal or death from any cause. Treatment beyond PD was allowed if there was a clinical benefit according to clinician’s decision.

### 2.5. Radiological Response Evaluation

Baseline radiological evaluations included a baseline total body computed tomography (TB-CT) scan, subsequently performed every 3–4 cycles or every 9–12 weeks as per standard of care, or whenever progression was clinically suspected. Six categories of radiological response were taken into consideration in this study to assess tumor response. Four of them (standard categories) were included in Response Evaluation Criteria in Solid Tumors (RECIST1.1): complete response (CR), partial response (PR), stable disease (SD), and progressive disease (PD). Two additional categories were included: hyper progression disease (HPD), an atypical pattern of response to single-agent IO (an acceleration of progression compared to the natural history of the disease) as defined by Ferrara et al. [22] and Lo Russo et al. [23], and eventually, not evaluable (NE) as the sixth category, comprising those patients who died due to PD before the first radiological evaluation.

## 3. Statistics and AI Methodology

**Figure 2:**
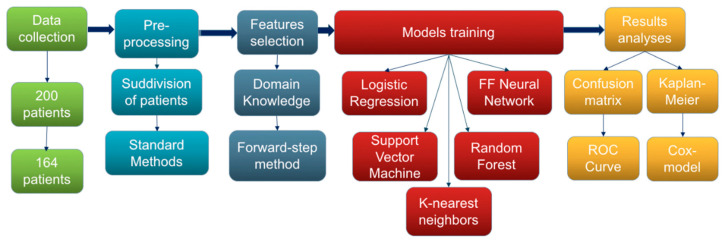
Process and methods used in this study.

Figure 2 reports an outline of all the methodologies applied for data analyses.

### 3.1. Statistical Analysis

Out of the 200 patients included in the present study having available PD-L1 expression data, 164 patients were used as the dataset for the ML algorithms, since PD-L1 was the only predictor used in clinical practice. Conversely, all 200 patients were included in the survival analysis. The first endpoint of the study was prediction of responder (R) and non-responder (NR) patients. The R group included patients who obtained a CR, PR, or SD as per RECIST 1.1, while the NR group included those patients who obtained a PD per RECIST1.1., or an HPD or NE response (as described above).

Other endpoints were at 24-months overall survival (OS), median progression-free survival (mPFS), and median OS (mOS). mOS was measured from the starting date of IO therapy until death, or last follow-up. mPFS was calculated from the starting date of IO until PD or death due to any cause, or last follow-up visit for alive patients without PD. Kaplan–Meier was used to calculate mPFS and mOS with their 95% confidence interval, and to generate survival curves. Cox’s proportional hazards model was used to calculate the hazard ratio (HR) between R and NR groups according to OS and PFS.

### 3.2. Machine Learning Methods

After data collection, descriptive analysis and data processing were performed. A first step consisted of the selection of a set of 21 features determined to be the most relevant based on the published literature on NSCLC patients treated with IO and clinician experience. Finally, in the case where a pair of features showed a linear correlation higher than 0.8, we removed one of them, as customary in ML studies. The result was the set of M = 15 most relevant features, provided in Table 1.

The problem of predicting R and NR was modelled as a binary classification problem, where we wanted to learn an approximation f ^(x_i) of the real relationship y = (x_i) between the i-th patient’s feature vector x_i and the response y_i ∊ {0,1}, where a patient has y_i = 0 for NR, and y_i = 1 for R. The same modelling was applied to the problem of estimating survival at 24 months, i.e., a patient has y_i = 0 for those patients with OS less than 24 months and y_i = 1 for those with more than 24 months. Data corresponding to the 40 alive patients with less than 24 months were excluded from this second analysis.

A set of appropriate techniques from the ML literature were selected to perform the above-mentioned classification task. More specifically, feedforward neural network (FFNN), logistic regression (LR), K-nearest neighbors (K-NN), support vector machines (SVM), and random forest (RF) were tested. A feature selection approach to select the proper subset of the original M features appropriate for each method were applied. More specifically, a forward feature selection using the AIC criterion as metric to select the most appropriate set of features for each method and the best method were used. The 5-fold cross-validation ACC and F1 scores for the analyzed methods, as well as the leave one out AUC, with the corresponding 95% confidence intervals were computed using the bootstrap approach (in brackets).

The procedure was implemented in Matlab, and the code performing all of the ML procedures is available at https://trovo.faculty.polimi.it/downloads.html (accessed on 10 October 2021) [24].

## 4. Results

### 4.1. Patients’ Characteristics

Two hundred NSCLC patients treated with anti-PD-(L)-1 in first or further-line therapy were included in the survival analysis. Most patients were male (65%) and smokers (79.5%), median age was 67 years (range 60–74 years), and 38% of patients were older than 70 years. PD-L1 was ≥50% in 53 (26.5%), 1–49% in 59 (29.5%), <1% in 52 (26%) and unknown in 36 (18%) patients. Median ECOG-PS was 1 (range 0–1) with an ECOG PS 2 in 14.5% of patients. All patients had a histological diagnosis of NSCLC (77% non-squamous and 23% squamous) and were epidermal growth factor receptor (EGFR) non-mutated and anaplastic lymphoma kinase gene (ALK) non-translocated. At baseline IO, liver metastases were present in 35 (17.5%) of patients. More than one-third of patients (35%) received IO in first line, while the remaining patients received anti-PD-(L)-1 therapy in further lines. Overall, 40 (20%) patients were H, 65 (32.5%) were I, and 54 (27%) were L according to MSC risk level. On the other hand, 41 (20.5%) patients were E and thus not analyzable.

One-hundred sixty-four patients were enrolled in this study, and patients were divided into two major groups: 73 belonged to the R group (CR, PR, or SD), and 91 to the NR group (PD, HPD, or NE).

### 4.2. Predicting Responder and Non-Responder Patients

Table 2 presents the results of the feature selection procedure. The best model turned out to be the logistic regression, which included 5 features: ECOG performance status, IO line of therapy, the neutrophil-to-lymphocyte ratio (NLR), the MSC test, and PD-L1. The importance of the variables was provided directly by the magnitude (absolute value) of the coefficient obtained by the logistic regression. More specifically, in order of importance for the LR, the parameter vectors learned by LR were w = 1.058 (NLR), 0.71 (IO line), 0.692 (ECOG), 0.566 (MSC), and −0.471 (PD-L1 > 50%). This showed how an increase in one of the first four features was negatively correlated with patients’ response, and conversely, how the increase in the PD-L1 value correlated positively with response (the only negative coefficient).

**Figure 3:**
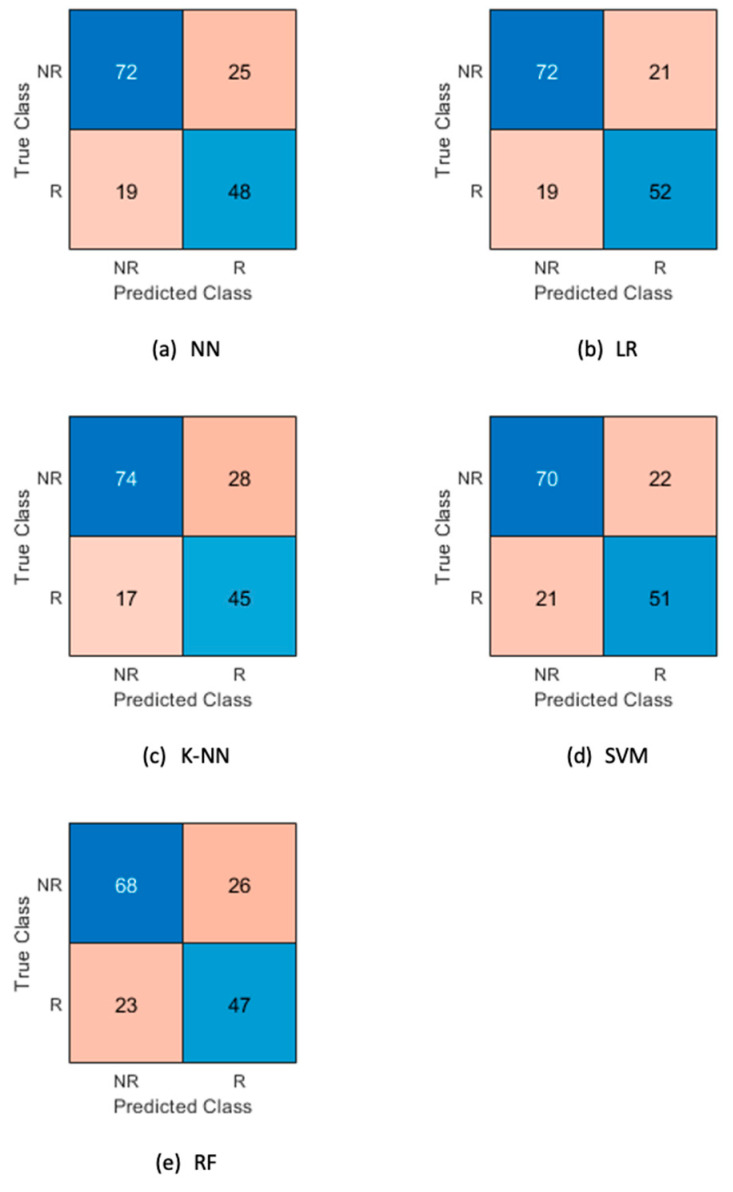
Confusion matrix for the analyzed ML models for responders (R) and non-responders (NR) for ML model algorithms (**a**) NN, (**b**) LR, (**c**) K-NN, (**d**) SVM and (**e**) RF.

For each model, the confusion matrix is presented in Figure 3 to show their performances in terms of true/false positives/negatives.

**Figure 4:**
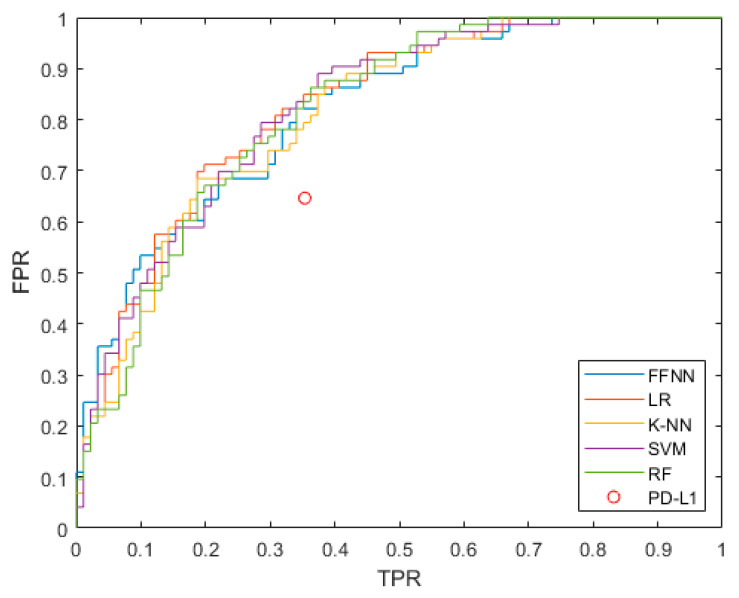
ROC curves (true positive rate (TPR) vs. false positive rate (FPR)) for the analyzed ML models. The performance of PD-L1 is represented as a red circle. As suggested by the AUC confidence intervals, there is no method that outperforms the others significantly.

**Figure A1:**
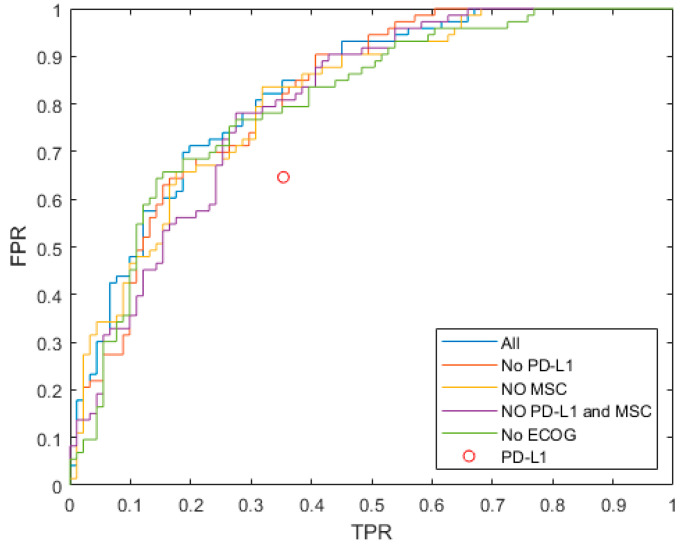
ROC curves for the LR method starting from different sets of features. The performance of PD-L1 is represented as a red circle. As suggested by the AUC confidence intervals, there is no method that outperforms the others significantly.

Logistic regression as the best model achieved an ACC = 0.756, F1 = 0.722, and AUC = 0.83. PD-L1 alone had an ACC = 0.655 (whose performances are illustrated by the red circle in Figure 4). We also evaluated the accuracy of the LR models excluding PD-L1, MSC, and both PD-L1 and MSC from the models, i.e., considering only clinical features. Moreover, we excluded the ECOG, being the only physician-dependent feature. The results of these models are shown in Table 3, and the ROC curves are provided in Figure A1. Removing PD-L1, the accuracy of the corresponding model decreased to ACC = 0.726, confirming the high importance of this feature, as reported in the literature. Removing the MSC from the feature decreased the accuracy to ACC = 0.750, suggesting that the predictive power of this index was less impactful than PD-L1. Removing both from the data yielded an ACC = 0.707.

Finally, removing the ECOG decreased the accuracy of the LR model to ACC = 0.726; therefore, the importance of the physician clinical evaluation was comparable to PD-L1 in the prediction. These findings were confirmed by the values of the F1 score and the average AUC (Table 3). The ROC curve obtained by the leave-one-out method is presented in Figure 4.

### 4.3. Survival Analysis According to PFS and OS

**Figure 5:**
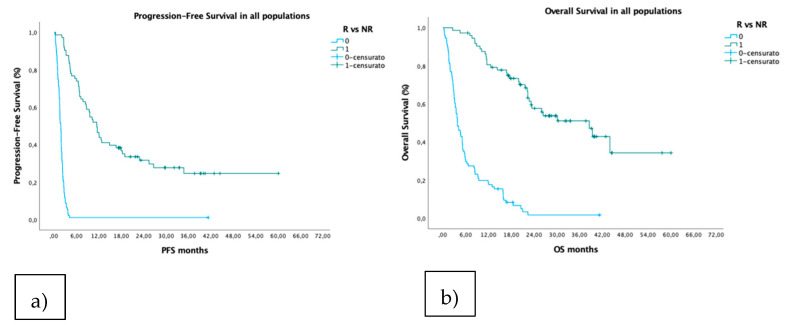
Kaplan–Meier according to PFS (**a**) and OS (**b**) curves divided by R (red curves) and NR (blue curves) groups.

**Figure A2:**
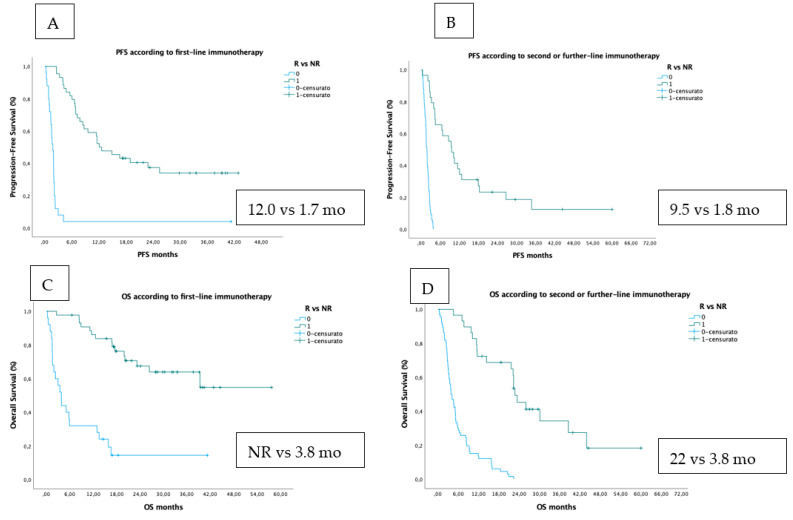
The Kaplan–Meier curves according to line of therapy in responder (R) and non-responder (NR) groups are reported. PFS for first (**A**) and second or further line (**B**) and OS for first (**C**) and second or further line IO (**D**).

Since good results were obtained in classifying patients as responders and non-responders, it was also possible to estimate the mOS and mPFS of these patients using KM curves, as shown in Figure 5a,b. At data cut-off (November 2020), mOS was 10.1 months for all patients. Median PFS for the R and NR groups was 11.4 vs. 1.8 months (HR 0.095, 95%CI 0.062–0.114, *p* < 0.0001), and the median OS, 38.5 vs. 3.8 months (HR 0.123, 95%CI 0.079–0.193, *p* < 0.0001). Appendix A contains all of the Kaplan–Meier curves separately according to first and further-line therapy in R and NR patients, respectively (Figure A2).

### 4.4. Predicting Long-Survival Patients (≥24-Months OS)

To predict long-survival (≥24-months OS) patients, another ML binary classification analysis was performed.

Because we were solving a different classification model, we needed to reconsider the use of the above-mentioned methods from scratch. Table 4 lists all of the procedures for feature selection. Even in this case, the LR method proved to be the most promising according to the AIC criterion. It achieved an ACC = 0.855, F1 = 0.908, and AUC = 0.87. The features included in the model were ECOG, histology, NLR, and IO line.

**Figure A3:**
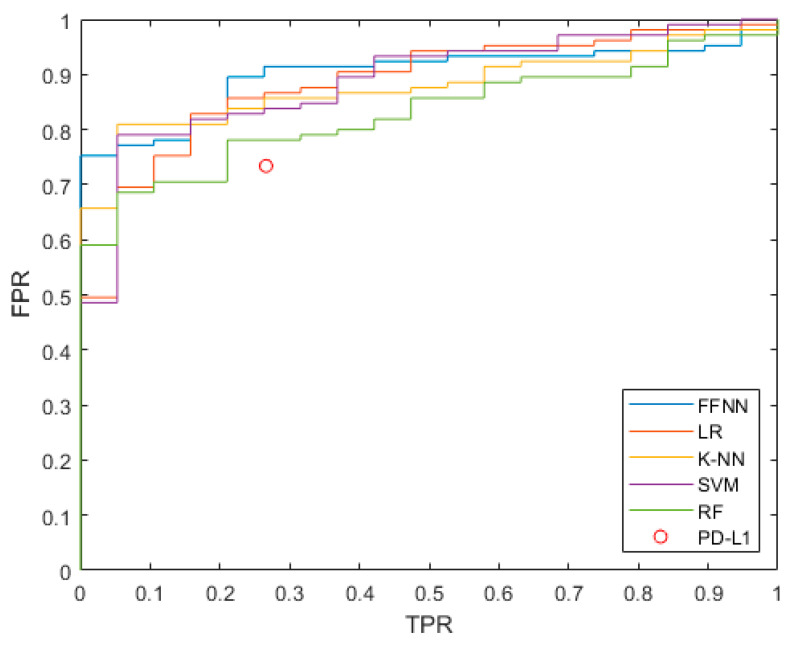
ROC curves for the LR method predicting the patients’ survival (longer or shorter than 24 months). The performance of PD-L1 is represented as a red circle.

The ROC curves computed using the leave-one-out approach are provided in Figure A3.

## 5. Discussion

The use of AI is attracting great interest in the medical field and, in particular, in oncology. The recent literature contains a wide range of publications regarding the use of AI applied to NSCLC, especially focusing on real-world data, genomics, circulomics, and radiomics. In our study, we aimed to find an algorithm to predict response to and efficacy of IO using real-world data (i.e., clinical, tumor, and treatment data) and translational data (i.e., the results of the MSC test). Combining the current medical literature, clinical experience of physicians, and ML tools, we developed an algorithm including five important features discriminating between R and NR patients with good accuracy (ACC = 0.756, F1 = 0.722, and AUC = 0.83). The model achieved significantly better results compared to PD-L1 prediction value alone, which is the only biomarker currently used by physicians in clinical practice to select NSCLC patients for IO with an accuracy of ACC = 0.655 on the analyzed dataset. To determine whether the algorithm maintained its accuracy using only real-world data, we decided to exclude the PD-L1 from the model features. In this case, the accuracy of the model decreased, suggesting that even if the PD-L1 alone is not enough to provide an effective response prediction, it remains an essential feature for IO prediction to be used in clinical practice. We did the same with the MSC, since this test is an expensive and time-consuming exam, and, therefore, its introduction in clinical practice needs to be justified. When we excluded the MSC from the model, the model accuracy decreased, albeit by less than in the case of PD-L1 exclusion, again suggesting that the MSC has a role in our model. We also tested the model removing the patient’s ECOG, which is a physician-dependent value, and the results demonstrated a significant impact, analogous to PD-L1. Since the model was able to discriminate between R and NR groups, we were also able to indirectly predict the PFS and OS of these patients.

With a binary classification approach, we provided a method to identify and predict those patients with long OS (≥24-months OS). Even in this case, the use of ML techniques showed a significant improvement over the use of PD-L1 (ACC = 0.855, F1 = 0.908, and AUC = 0.87 vs. ACC = 0.734).

Various papers have been recently published to address the same unmet clinical need not only in NSCLC but also in other different cancer types.

Radiomics features are frequently used to predict IO response in NSCLC patients. In the study by He et al. [25] with a dual propose, radiomics were applied to build a TMB signature. CT images were used to discriminate between high-TMB and low-TMB in 327 patients. The model was then applied to the IO of 123 patients’ dataset to evaluate risk stratification. The TMB radiomic signature reached an AUC of 0.74 [5]. The prediction was slightly lower compared to our study, probably indicating that the clinical features and patients’ presentations have comparably high relevance as tumor features and that it is important to consider them in the model.

Khorrami et al. [26] compared changes (“delta”) in the radiomic texture of CT scan patterns (139 patients) and associated them with tumor-infiltrating lymphocyte (TIL) density in diagnostic biopsies from 36 patients. A linear discriminant analysis classifier yielded an AUC of 0.88 ± 0.08 in distinguishing R from NR patients when CT scan features were combined with TIL density. However, 36 patients were included in this coupled analysis, and even if our study achieved a lower AUC, our model included four real-world datasets that were easier to be obtained compared to radiomics and TIL analysis.

Yang et al. [27] used 200 patients to develop a deep learning (DL) model integrating different data sources (serial radiomics, CT scans, laboratory and baseline clinical data) to identify R and NR subgroups to IO in NSCLC patients. The model reported an AUC of 0.80 (95%CI: 0.74–0.86), showing a smaller than expected value when compared to ours (AUC 0.82). A very interesting study called DeePaN [28] used a deep patient graph convolutional network to investigate the IO benefit in NSCLC patients. By integrating real-world data (age, sex, race, histology, stage, ECOG score, smoking status and previous treatment, blood analyses) and genomics in 1937 patients, the algorithm was able to divide patients into two different subgroups: beneficial and non-beneficial patients with an mOS of 20.35 and 9.42 months, respectively. Even though our sample was smaller, our model was able to predict survival and response with comparable results. The model also demonstrated the positive role of TMB and KRAS mutation in IO patients [28]. The study by Tian et al. [29] had a dual purpose: first, to predict a PD-L1 signature (PD-L1ES) using CT images (in 939 patients), and second, to predict IO response in NSCLC patients combining PD-L1ES and clinical features (in 77 patients). PD-L1ES was able to distinguish patients with a better PFS compared to those with a lower PFS. However, results of the combined model (PD-L1ES and clinical data) were superior to both the clinical and PD-L1ES models alone [29]. Our study also confirmed the importance of PD-L1 and the value it added to clinical features.

The development and validation of a 12-gene immune relevant prognostic signature for lung adenocarcinoma through ML strategies was investigated in 954 patients to predict IO. From a discovery dataset of 204 observations including microarray data of gene expression of 1811 genes, Cox regression was used to decrease the number of features to 336. Random forest was then used to extract the final 12 selected genes used to compute the risk score. Patients were classified into high- or low-score with an AUC of 0.854 (95%CI = 0.79–0.92). Patients with a high-risk score experienced lower survival comparing to those with the low-risk score (HR = 10.6, 95%CI = 3.21–34.95, *p* < 0.001) [30].

Independently from IO, ML and DL techniques are now used in research to predict NSCLC prognosis for patients treated with different therapies to better address precision medicine; however, these techniques are still far from their introduction in clinical practice. An interesting study used DL to implement OS prediction in NSCLC patients by integrating microarray and clinical data. A list of 15 relevant genes was built using seven known relevant biomarker genes and eight other less-known genes. Expression data on the 15 genes and the clinical data were combined and used to develop an integrative deep NN predicting the 5-year survival status of NSCLC patients with high accuracy (AUC: 0.8163, accuracy: 75.44%); these data were consistent and comparable with our results [31]. Another study developed an algorithm to predict NSCLC survival time in 1000 patients treated with different types of therapies. Thirteen features were included in the algorithm, e.g., number of primaries, tumor size, age, and stage. Random forest was the best model to predict short-term survival period (<6 months) [32].

Finally, IO biomarker prediction, as we mentioned above, is an unmet clinical need also for other cancer types. In fact, as in NSCLC, various efforts have been made to find predictive biomarkers of IO response using ML or DL methodology in other cancers. An interesting report on melanoma patients integrated histologic data and clinical data to predict IO response. The algorithm consisted of a segmentation classifier that took as input the whole slide image of the patient (hematoxylin and eosin tissue). These results were then combined through a multivariable logistic regression with clinical characteristics such as age, gender, histologic subtype, etc. The classifier accurately stratified patients into high versus low risk for disease progression with an AUC = 0.80 [33].

Gene expression data were used to separate gastric metastatic cancer patients into durable clinical benefit (DCB) and non-durable clinical benefit (NDCB) groups considering a training dataset of 25 (DCB) plus 45 (NDCB) and a validation cohort of 9 (DCB) plus 15 (NDCB), obtaining an accuracy of ACC = 0.857 in the validation cohort [34].

Lastly, in another work regarding IO prediction in bladder cancer, CT-scans were used to develop an ML model according to the RECIST methodology, and the ROIs were processed to extract radiomic features. Considering a dataset of 43 subjects, the model reached an accuracy of ACC = 0.861 [35].

Our study had various limitations: firstly, the limited sample size. Secondly, we did not use radiomic features in our study, and no genomic data were included except the unique molecular data requested for standard of care.

Many studies have sought to extract more information from imaging (radiomics) and genomic data. Radiomics is a very important frontier but still in an early phase, and more time will be needed to include it in clinical practice. The same may be said for genomics. The approach adopted in this paper used routine information from imaging (e.g., RECIST) as well as real-world genetic data that had already been investigated as per standard of care, both of which added to the clinical information and enabled better extraction of predictive multifactorial information. These data can also be less expensive and easier to collect.

## 6. Conclusions

In conclusion, the results suggest that the data integration provided by AI techniques is a good tool to improve prediction for NSCLC patients treated with IO. More specifically, the model showed that higher ECOG, NLR value, IO line, and MSC test level correlated negatively with the response to IO therapy, whereas conversely, higher PD-L1 correlated positively with the response. It also confirmed that PD-L1 and MSC were relevant biomarkers to improve the accuracy of the model. Moreover, considering the difference in survival among R and NR groups, these results suggest that the model could also be used to indirectly predict survival (PFS and OS).

Finally, a second binary model was able to identify long survival patients with high accuracy.

## Figures and Tables

**Table 1:** Features selected based on literature review and clinician experience, and keeping only one of the variables in a pair showing linear correlation >0.8.

Feature Classes	Features
Clinical features	Age, sex, smoker/non-smoker, packs per year, ECOG
Laboratory exams	NLR * NLR4, LDH
Tumor features	PD-L1, histology (adenocarcinoma, squamous, other)
Radiological	Metastatic sites (liver, brain, bone)
Treatment features	IO line (first or further line)
Omic features	MSC test

* NLR was used both as a continuous variable or binary variable with cut-off 4.

**Table 2:** Features selected for the different models and corresponding performances.

ML Model	Selected Features	AIC	ACC	F1	AUC
LR	ECOG, IOLine, NLR, MSC, PD-L1	132.5	0.756	0.722	0.83 (0.76–0.88)
FFNN	NLR, IOLine, MSC, LDH, ECOG, PackYear	137.2	0.732	0.686	0.80 (0.73–0.86)
K-NN	NLR, IOLine, ECOG, MSC, NLR4	137.4	0.726	0.667	0.81 (0.74–0.87)
SVM	ECOG, IOLine, NLR, MSC, PD-L1	134.5	0.738	0.703	0.83 (0.75–0.88)
RF	NLR, IOLine, ECOG, Age, MSC	135.5	0.701	0.657	0.82 (0.73–0.87)

**Table 3:** Performances of the LR method when some of the features are removed from the initial pool of available ones.

Initial Feature Set	Selected Features	ACC	F1	AUC
All	ECOG, IOLine, NLR, MSC, PD-L1	0.756	0.722	0.83 (0.76–0.88)
No PD-L1	ECOG, IOLine, NLR, MSC	0.726	0.696	0.82 (0.75–0.88)
NO MSC	ECOG, IOLine, NLR, PD-L1, Age	0.750	0.709	0.81 (0.74–0.87
NO PD-L1 and MSC	ECOG, IOLine, NLR, Age	0.707	0.662	0.80 (0.73–0.86)
NO ECOG	IOLine, NLR, MSC, PD-L1	0.726	0.690	0.80 (0.73–0.87)

**Table 4:** Features selected for the different models and corresponding performances for the task of predicting the long- survival patients.

ML Model	Selected Features	AIC	ACC	F1	AUC
LR	ECOG, Histology, NLR, IOLine	58.1	0.855	0.917	0.89 (0.80–0.94)
FFNN	Histology, NLR, PD-L1, NLR4	61.4	0.839	0.908	0.87 (0.78–0.92)
K-NN	NLR, PD-L1, Histology	60.6	0.847	0.916	0.88 (0.80–0.93)
SVM	Age, Histology, MSC, ECOG, PD-L1, NLR	63.2	0.847	0.913	0.90 (0.83–0.94)
RF	NLR, PD-L1	63.8	0.847	0.917	0.83 (0.74–0.89)

## Data Availability

Data are available from the authors upon reasonable request. The code for all the ML procedures is available at https://trovo.faculty.polimi.it/downloads.html (accessed on 10 October 2021).

## Abbreviations

Non-small cell lung cancer (NSCLC), immunotherapy (IO), programmed death-ligand 1 (PD-L1), tumor mutational burden (TMB), tumor microenvironment (TME), microRNA (miRNA), artificial intelligence (AI), machine learning (ML), plasma microRNA signature classifier (MSC), quantitative reverse transcription PCR (RT-qPCR), low (L), intermediate (I), high (H), hemolyzed (E), intravenously (IV), weeks (w); total body computed tomography (TB-CT), response evaluation criteria in solid tumors (RECIST1.1), complete response (CR), partial response (PR), stable disease (SD), progressive disease (PD), hyper progression disease (HPD), responder (R), non-responder (NR), median progression-free survival (mPFS), median overall survival (mOS), hazard ratio (HR), Akaike information criterion (AIC), neutrophil to lymphocyte ratio (NLR), lactate dehydrogenase (LDH), accuracy (ACC), area under the curve ROC (AUC), tumor-infiltrating lymphocyte (TIL).
